# Treating winter depressive episodes in bipolar disorder: an open trial of light therapy

**DOI:** 10.1186/s40345-020-00182-5

**Published:** 2020-06-01

**Authors:** Lotte J. E. van Hout, Lisette E. P. Rops, Claudia J. P. Simons

**Affiliations:** 1 GGzE, Institute for Mental Health Care Eindhoven, Dr. Poletlaan 40, 5626 ND Eindhoven, The Netherlands; 2 Department of Psychiatry and Psychology, School of Mental Health and Neuroscience, Maastricht University Medical Center, Maastricht, The Netherlands

**Keywords:** Light therapy, Bipolar disorder, Seasonal affective disorder, Winter depression

## Abstract

**Background:**

Light therapy has been used to treat winter depression in bipolar disorder, although the dose, duration, and timing of treatment have differed. The present study is an open trial of light therapy for depressive episodes in autumn/winter using a Dutch protocol specific for patients with a bipolar disorder.

**Methods:**

Data were collected for the seasons September–April 2017–2018 and September–April 2018–2019. In total, 58 patients received light therapy for a minimum of 7 days and a maximum of 21 days; there was a follow-up measurement after two weeks. Outcomes were quick inventory of depressive symptomatology (QIDS) scores and side effects.

**Results:**

QIDS scores were significantly lower at the last day of therapy (B = − 6.00, p < 0.001) and 2 weeks after the end of treatment (B = − 6.55, p < 0.001) compared with pre-intervention. Remission (QIDS ≤ 5) was reached in 55% of the treatments and response (50% symptom reduction) in 57% of the treatments. Side effects were mild; two hypomanic periods occurred.

**Conclusions:**

The Dutch light therapy protocol for patients with a bipolar disorder may be effective in treating a seasonal depression and side effects are mild. Light therapy deserves a prominent place in the treatment because effects may be large and quick.

## Introduction

For decades, light therapy has been an effective method for the treatment of seasonal affective disorder and non-seasonal affective disorder (Rosenthal 1984; Kripke 1998; Ruhrmann et al. 1998; Tuunainen et al. 2002; Golden et al. 2005). Since the first article on this subject in 1984 by Rosenthal (1984), research has focused on the optimization of the treatment for these groups of patients. For a long time, patients with a bipolar disorder were excluded from this research because of fear that the therapy can induce a (hypo)manic state.

In the last decade, however, research has shown that there is a remarkable resemblance between the pathophysiology of seasonal affective disorder and a bipolar disorder (Geoffroy et al. 2014, 2015). It has even been suggested that 11–50% of the patients with a seasonal depression actually can be diagnosed with a bipolar disorder (Sohn and Lam 2004). This, in combination with low response rates for antidepressants and their potential risk of inducing a (hypo)manic state, prompted clinical studies to investigate the effects and risks of light therapy for bipolar depression (Zhang et al. 2013; Goldberg and Truman 2003; Tondo et al. 2009).

However, research on these effects is still limited and results regarding the effectivity are inconclusive. In their meta-analysis, Tseng et al. (2016) found a significant decrease in severity of bipolar depression after light therapy. In 2018, Sit et al. (2018) found that patients with a bipolar depression treated with bright white light had a significantly higher remission rate and lower depression scores compared to a placebo group. Dauphinais et al. (2012), however, found no evidence for a difference between light therapy and placebo. A recent study of Zhou et al. (2018) showed a positive effect of light therapy in comparison to a control group in patients with a bipolar depression. A possible explanation for the mixed findings may be that previous studies differed in the way light therapy was given. There are, for example, differences in length of the treatment, time of day of receiving light therapy, the amount of lux, the type of light, the exposure time, and the combination with other interventions (Sit et al. 2018; Dauphinais et al. 2012; Zhou et al. 2018; Wu et al. 2009; Knapen et al. 2014; Dimitrova et al. 2017; Suzuki et al. 2018; Meesters et al. 2018; Kupeli et al. 2018).

Looking at the risk for developing a (hypo)manic state, a meta-analysis by Benedetti (2018) shows a 0.9% risk for a switch into mania and a 1.4% risk for a switch into hypomania. In comparison, the switch rate during placebo treatment was estimated at 4%. Although these low switch rates are reported, light therapy is not used in common practice.

Because of the potential that light therapy has, and since the risk of developing a (hypo)manic state is considered limited, the Dutch Chronotherapy Comity of the national center for bipolar disorders (Kenbis) wrote a treatment protocol (Eldering et al. 2018). This protocol was optimized for safety, effectiveness and clinical practicality in treating patients with a bipolar depression.

### Aim of the study

The aim of this study was to evaluate the safety and effectiveness of light therapy for treating depressive symptoms in patient with a bipolar disorder using the Dutch protocol. This study reflects on the results of light therapy focusing on the effectiveness and the risk of developing a (hypo)manic state in patients with a bipolar depression. The Dutch protocol of light therapy for bipolar disorders was followed for two consecutive years in the autumn/winter at the center for bipolar disorders Eindhoven, the Netherlands.

## Methods

### Treatment setting

Data were collected at the Center for Bipolar Disorders of GGzE, Eindhoven, the Netherlands. This center is an outpatient clinic specialized in the treatment of bipolar disorders for adults. Patients receive pharmacological, psychological, and/or supportive care.

All data were collected retrospectively, as light therapy was offered as standard care. Written consent was not collected since all data were collected for clinical purposes. Patients had the opportunity to refuse the use of their anonymized data for scientific research. The institutional review board of GGzE gave ethical clearance for the retrospective, anonymized use of the clinical data for research purposes and provided a waiver of informed consent. Identifying information was not provided to the researchers outside of the treatment team.

### Indication for light therapy

All patients of the treatment facility who were diagnosed with a bipolar disorder (type I or II) and experienced depressive symptoms in the period September 2017–March 2018 and/or September 2018–March 2019 were offered light therapy at our center. Light therapy was recommended as therapy of first choice but participation was not obligated, no changes in medication were made right before or during the light therapy.

A current (hypo)manic or mixed state was considered a contra-indication for light therapy. Based on theoretical considerations, no contra-indications regarding ocular diseases, medical conditions, or medication use that influence photosensitivity seem to be indicated (Brouwer et al. 2017). If there was any doubt, an ophthalmologist could be consulted to check if light therapy was safe. Photo-sensitive medication also includes lithium. Because clinical studies did not find a negative effect of long-term lithium use on the retina, use of lithium was not seen as a contra-indication for the use of light therapy (Lam et al. 1997).

### Screening protocols

If patients experienced depressive symptoms and there were no contra-indications for treatment with light therapy, the severity of depressive symptoms was assessed with the quick inventory of depressive symptomatology (QIDS). The QIDS is a questionnaire containing 16 questions rating the nine symptom domains defining a depressive episode according to the DSM-IV. Symptoms are rated for the prior seven days (Rush et al. 2003; Bernstein et al. 2010). A QIDS score of six or higher indicated a depression and was reason to start light therapy (Rush et al. 2003; Bernstein et al. 2010; Trivedi et al. 2004; Maaren et al. 2013; Meesters et al. 2016).

### Treatment protocol

Patients were treated with an intensity of 10,000 lx for at least seven sequential days. On weekdays, light therapy was given with a Davita PhysioLight LD 220 lamp with a strength of 10,000 lx. Distance to the lamp was 50 cm. Screen size of this device is 60 × 40 cm. This lamp generates white light with a light filter that reduces the energy of light emitted in the infrared and UV range to a minimum. Patients were instructed to sit in front of the lamp for 30 min. Patients could also opt to take a lamp (multiple manufacturers, all 10,000 lx) home instead of coming to the treatment center. In the weekends, all patients received these types of lamps to use at home. Patients were instructed about the use of and the distance to these lamps as well (30 cm). Light therapy was conducted between 8:00 a.m. and 11:00 a.m., as several studies suggest that light therapy in the morning has been proven more effective than light therapy in the mid-day (Tuunainen et al. 2002; Golden et al. 2005; Eastman et al. 1998; Terman et al. 1998). At the start, after scoring the QIDS, patients were instructed by a clinical professional on the correct use of the lamp.

After 1 week, patients had an appointment with a clinical professional to evaluate the light therapy, including an assessment of depressive symptoms using the QIDS and an evaluation of the adverse effects of the light therapy. The Young Mania Rating Scale (YMRS) was scored if there was a suspicion of a switch to a (hypo)manic state *or a mixed state* based on the clinical presentation or on the anamnestic complaints of the patient (Young et al. 1978). The score together with the clinical impression of the clinical professional was used to come to the conclusion whether light therapy could be continued or was stopped. If patients had a QIDS ≤ 5, indicating remission, light therapy stopped. If the QIDS score was > 5, patients received seven more days of light therapy with the same treatment conditions as the first week. After this week (i.e. after 14 days of treatment), the QIDS was scored for the third time. Again, if the QIDS score was five or lower, remission was reached and the treatment was stopped. If not, another week of light therapy was offered. After a maximum of 21 days, the treatment stopped even if remission was not yet reached. Fourteen days after light therapy ended, a follow-up assessment with the QIDS was conducted. This was to see if the effects remained after light therapy ceased.

### Statistical analysis

As QIDS observations were clustered within light therapy interventions, within patients, we conducted multilevel mixed effects regression analyses with light therapy intervention and patients as random effect (i.e., random intercepts). The QIDS score after the last light therapy session (either at day 7, 14, or 21) was used as the post score. The QIDS score 14 days after the last light therapy session was the follow-up score. The regression models were run with QIDS total scores as dependent variable and session (model 1: 0 = pre or 1 = post; model 2: 0 = pre and 1 = follow-up) as dependent variable. The model was fitted with the State mixed command (Stata version 13; StataCorp) using restricted maximum likelihood estimation (reml). Remission and response were calculated, as they provide information on the clinical relevance of the effects. A QIDS score < 6 was seen as remission of the depressive episode. Response was defined as a reduction of ≥ 50% on the QIDS. In addition, QIDS severity was established using the cut-off scores defined by Rush et al. (2003).

## Results

We collected data from 58 patients with 67 treatment periods (some patients had more than one treatment period). In total, we collected 237 QIDS observations. For demographic information, see Table 1. A total of 67 treatment periods (in 58 patients) were analysed. At day 7, 15 treatment periods let to remission (22%), two treatment periods (3%) were stopped before day 7. At day 14 another 15 treatment periods (22%) scored below 6 indicating remission. At the end of the three weeks of treatment another 5 treatment periods let to remission (7%). A total of 14 treatment periods stopped before the three weeks of treatment was completed (21%). Sixteen treatment periods did not lead to remission of symptoms even though the three weeks of treatment were completed (24%).

**Table 1:** Patient characteristics (n = 58 patients)

	N	%
Female	36	62.1
Male	22	37.9
Diagnosis		
Bipolar I	36	62.1
Bipolar II	22	37.9
Medication^a^		
Mood stabilizers^b^	47	
Lithium	33	
Valproic acid	9	
Carbamazepine	1	
Lamotrigine	4	
Antipsychotics	33	
Anti-depressant^c^	29	
Sedatives^d^	25	
None	3	
Severity depression^e^		
Mild	19	28.4
Moderate	26	38.8
Severe	19	28.4
Very severe	3	4.5
Age	M: 47.6; SD: 14.05; Range: 20–78	

^a^ Note that no percentages were given because patients could use more than one medication

^b^ Lithium, valproic acid, carbamazepine, lamotrigine

^c^ Selective serotonin reuptake inhibitor (SSRI), serotonin-norepinephrine reuptake inhibitor (SNRI), noradrenergic and specific serotonergic antidepressant (NaSSA), tricyclic antidepressant (TCA)

^d^ Sedatives: benzodiazepines, melatonin, Z-drugs, Levomepromazine

^e^ According to the QIDS cut off points (Rush et al. 2003)

At follow-up, two weeks after light therapy, patients were asked to participate in another QIDS interview, 37 patients replied. Sixteen patients did not complete the light therapy protocol; the main reason seemed to be remission of the complaints (in between the moments of scoring the QIDS) or a lack of effect.

Table 2 presents the QIDS scores at different assessment moments. Multilevel regression analysis revealed that the total QIDS scores at post-assessment (final light therapy session at day 7, 14, or 21) were significantly lower than the QIDS scores at pre-assessment [*B* = − 6.00, 95% CI − 7.16; − 4.85; *χ*^*2*^(1) = 103.68, *p* < 0.001]. Two weeks after the final light therapy session, QIDS scores were still significantly lower compared with pre-light therapy scores [*B* = − 6.55 95% CI − 7.97; − 5.13, *χ*^*2*^(1) = 81.58, *p* < 0.001].

**Table 2:** Mean depression score according to the quick inventory of depressive symptomatology (QIDS)

	Number	Mean	Standard deviation	Minimum score	Maximum score
Day 0	67	13.18	4.41	6	24
Day 7	64	9.31	4.87	0	20
Day 14	47	8.55	5.24	1	24
Day 21	22	9.68	5.91	3	23
2 weeks follow-up	37	5.97	4.82	0	20

**Fig. 1:**
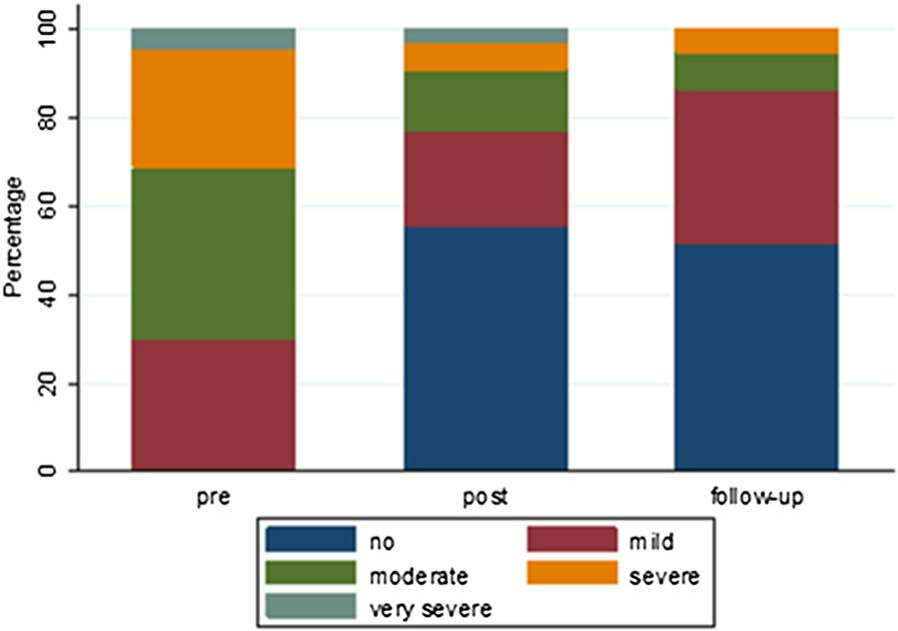
Severity of depressive symptoms, categorised based on QIDS scores

We found that 55% of the completed treatments led to remission (QIDS ≤ 5 at day 7, 14 or 21); 57% led to a response (response defined as a 50% decrease of initial QIDS score at day 0). The distribution of the severity of depressive symptoms per assessment can be found in Fig. 1.

### Adverse effects

Side effects were also monitored during the study. Side effects reported were: nausea (n = 1), headache (n = 1), and feelings of agitation and restlessness (n = 3). These complaints dissolved quickly after the daily dose of light therapy was given and no deviation of the amount of lux or length of the light therapy was needed.

The YMRS interview was conducted once due to suspicion of manic symptoms. Although the patient quitted therapy because of a feeling of restlessness and a decrease in sleep, the YMRS score of 4 indicated that the symptoms did not meet criteria for a (hypo)manic state. Two other patients, however, did switch to a (hypo)manic state, as indicated by notes in the patient file. One patient was admitted because of a hypomanic state seven days after the end of light therapy. After a brief admission with optimisation of the medication, the hypomanic state was in full remission. The other patient was treated with an increase of the dosage of lithium for one week with full recovery after one week. Thus 2.99% of the treatments (i.e., in 3.45% of the patients) led to a hypomanic state.

## Discussion

Where light therapy has been a commonly used treatment method for seasonal affective disorder, the use of light therapy for treating patients with a bipolar disorder stayed behind. The current study examined the effectiveness and safety of light therapy for the clinical practice according to the Dutch protocol for light therapy for bipolar disorders.

This study is the first to examine the effect of light therapy in the clinical practice using this treatment protocol and shows positive results with regard to the effectivity of light therapy in treating depressive periods in patients with a bipolar disorder. More than half of the treatments led to remission (55%) and/or response (57%) at the last day of light therapy. There was a significant decrease in QIDS score at the last day of therapy (B = − 6.00, p  < 0.001) and two weeks after the end of treatment (B = − 6.55, p  < 0.001). This suggests that light therapy may decrease the severity of depressive symptoms during the treatment and shows that the decrease in depressive symptoms persisted after light therapy was ceased. The quick effect of light therapy is in line with previous studies (Tuunainen et al. 2002; Golden et al. 2005; Terman and Terman 2005). This is an advantage in comparison with anti-depressants or mood-stabilizers, which take more time to show an effect. There is some evidence that light therapy combined with sleep deprivation can be even more effective (Wu et al. 2009), but because of the great impact on daily life of sleep deprivation and the efficacy of light therapy without sleep deprivation, treatment with just light therapy can be a more suitable option for many patients.

An important aim of our study was also to establish the safety of the Dutch light therapy protocol. In the review of Benedetti (2018), the highest reported risk of a (hypo)manic decompensation as a result of light therapy was close to 4%. Similarly, in our study 2.99% of the treatments led to a hypomanic decompensation. No manic decompensation was reported. This supports light therapy is a safe treatment option for this group of patients.

Side effects that were reported in the current study were nausea, headache and a feeling of agitation. This is comparable to previous studies that reported insomnia, headaches, irritability, and dizziness (Dauphinais et al. 2012; Zhou et al. 2018; Kupeli et al. 2018; Levitt et al. 1993; Labbate et al. 1994; Terman and Terman 1999).

The current findings thus suggest that light therapy administered in the morning may be effective in treating winter depression in bipolar disorders. It should be noted however, that the best timing of light therapy has been debated given that light therapy has also been shown to be effective in patients with seasonal depression when administered in midday of evening (Wirz-Justice 1993; Terman et al. 1998). Although several studies suggest that mornings are the optimal timing for light therapy (2002; Golden et al. 2005; Eastman et al. 1998; Terman et al. 1998), the studies by Sit et al. (2007, 2018) advocate light therapy in midday for patients with a bipolar disorder as light therapy may lead to (hypo)mania when administered in morning. The current findings and the review by Benedetti (2018) dispute this, however, and suggest that the risk of switching to mania was not increased after morning light therapy.

This altogether shows that the current light therapy protocol may be effective and safe for treating depressive symptoms in autumn/winter for patients with a bipolar disorder. To strengthen this conclusion, a randomized controlled trial is indicated as the next step. Further research should aim to investigate the predictors for a successful treatment. Also, more research is needed to examine the effectiveness of this treatment in spring and summer.

## Limitations

A major limitation of the study is that the data were collected as part of standard care and, hence, there was no control group. We cannot rule out the possibility that, e.g., the depressive symptoms decreased simply as an effect of time or as a placebo effect. Studies in the past showed that the expectations of patients have a significant impact on the antidepressant effect, (Rutherford et al. 2012) thus we cannot rule out that expectations explain a significant part of the effect we found. However, given the short treatment period, the severity of the symptoms, and the population (patients in specialized care with recurrent depressive episodes), we regard it unlikely that the present effects can merely be explained by spontaneous recovery of the depressive symptoms.

Unfortunately, we were not able to see if there was a difference between light therapy at home or at our out-patient clinic. We, therefore, cannot exclude the possibility that effects were (partially) driven by the activation and mobilization that it requires to get out of bed every morning to visit our center. However, previous studies that compared light therapy with a placebo control group also suggest that light therapy is effective in reducing depressive symptoms in patients with a bipolar disorder (Sit et al. 2018; Zhou et al. 2018; Wu et al. 2009; Suzuki et al. 2018; Kupeli et al. 2018; Benedetti et al. 2005).

Other limitations were the relatively small sample size and the fact that the raters were not blinded. Furthermore, the follow-up measurement at two weeks after the end of the light therapy was filled in by 55% of the patients. This means the results of the follow-up measurement must be carefully interpreted, as it is a possibility that the QIDS was predominantly filled in by people with positive results. Compliance with the use of the lamps, especially for the patients who took a lamp home, could not be assessed. This may have led to an underestimation of the treatment effect. This study evaluated the effects of a fixed-dosage protocol in which duration of the light therapy or the amount of lux given was not adjusted based on clinical response or adverse effects. For further research, it may be interesting to see if it is possible to create a more specific treatment protocol for every patient according to the effects and side effects of the treatment.

Finally, to detect potential (hypo)manic decompensation, the clinical observations of the well-trained staff were used. This lead to the discretionary administration of the YMRS, which risks under-detection of (hypo)manic symptoms.

## Conclusion

The results of this study show a decline in depressive symptoms after treatment with light therapy for patients with a bipolar disorder with depressive complaints in autumn/winter. The decrease in symptoms may still be observed two weeks after treatment. Risk of developing a (hypo)manic state appears limited and side effects were rare and mild. These findings suggest that the Dutch protocol for light therapy for patients with a bipolar disorder may be effective in ameliorating depressive symptoms and is safe. A randomized, controlled trial would be the next step to make this conclusion more definite.

## Data Availability

The datasets used and analysed during the current study are available from the corresponding author on reasonable request.
